# Mental health and its influencing factors of maintenance hemodialysis patients: a semi-structured interview study

**DOI:** 10.1186/s40359-023-01109-2

**Published:** 2023-03-28

**Authors:** Junjun Wen, Yuan Fang, Zhongyan Su, Jimin Cai, Zhiyan Chen

**Affiliations:** 1 CAS Key Laboratory of Mental Health, Institute of Psychology, Chinese Academy of Sciences, 16 Lincui Road, Chaoyang District, Beijing, 100101 China; 2 Department of Psychology, University of Chinese Academy of Sciences, Beijing, 101408 China; 3 Hemodialysis Center, Zhanlanlu Hospital, Beijing, 100044 China

**Keywords:** Maintenance hemodialysis, Mental health, Grounded theory, Semi-structured interview, Acceptance of disease

## Abstract

**Background:**

Maintenance hemodialysis (MHD) is a commonly used renal replacement therapy for end-stage renal disease patients. MHD patients have undergone multiple physiological stressors, which may cause physical problems and affect their mental health; however, few qualitative studies have been done on the mental health of MHD patients. Such qualitative research becomes the basis for further quantitative research and is critical to validating its results. Therefore, the current qualitative study used a semi-structured interview format, and aimed to explore the mental health and its influencing factors of MHD patients who are not receiving intervention treatment to determine how best to ameliorate their mental health.

**Methods:**

Based on the application of Grounded Theory, semi-structured face-to-face interviews were conducted with 35 MHD patients, following consolidated criteria for reporting qualitative studies (COREQ) guidelines. Two indicators (emotional state and well-being) were used to assess MHD patients’ mental health. All interviews were recorded, after which two researchers independently performed data analyses using NVivo.

**Results:**

Acceptance of disease, complications, stress and coping styles, and social support were found to be the influencing factors of MHD patients’ mental health. High acceptance of disease, healthy coping styles, and high social support were positively correlated with mental health. In contrast, low acceptance of disease, multiple complications, increased stress, and unhealthy coping styles were negatively correlated with mental health.

**Conclusion:**

One’s acceptance of the disease played a more significant role than other factors in affecting MHD patients’ mental health.

## Background

Chronic kidney disease (CKD) is a public health problem worldwide, with incidences of end-stage renal disease (ESRD) increasing yearly [1, 2]. As the most commonly used renal replacement therapy (RRT) for ESRD, maintenance hemodialysis (MHD) is in increasing demand by patients [3]. In RRT, MHD patients must receive continuous treatment sessions at a specific hemodialysis center at fixed time intervals. Conventional treatment generally takes place 2 to 3 times a week, with each session lasting 3 to 4 h [4]. If they are not able to adopt a different RRT method, such as kidney transplant or peritoneal dialysis, MHD patients require lifelong hemodialysis treatments [5]. Therefore, these patients must make lifestyle changes including dietary adjustments, strict control of water intake, regular application of hemodialysis-related drugs, and exercise training interventions [6–8]. Furthermore, loss of appetite [9], fatigue [10], pruritus [11], muscle weakness [12], and vascular access complications [13] may also occur in MHD patients undertaking long-term hemodialysis. All these complications can affect a patient’s quality of life (QOL) [14, 15] as well as their mental health. MHD patients experience different degrees of acceptance of their own disease and hemodialysis treatment. After undergoing lifestyle changes, experiencing and understanding the disease itself, and facing family and economic burdens, many MHD patients experience depression and anxiety symptoms [16], cognitive dysfunction [17], alexithymia [18], and other mental health problems.

The World Health Organization defined mental health as “a state of complete physical, mental, and social well-being, not merely the absence of disease or infirmity” [19]. Depression, anxiety, and negative emotions are used widely as indicators of negative mental health [20, 21]. Well-being, life satisfaction, and positive emotions are deemed as indicators of positive mental health [22–25].

Previous quantitative studies on the mental health of MHD patients have focused mainly on the prevalence of depression and anxiety symptoms, and on corresponding intervention methods such as drug therapy, cognitive behavioral therapy, and complementary therapy [26–30]. Meanwhile, few qualitative studies on this topic exist, despite qualitative research being the basis of quantitative research and critical to validating the results of quantitative research, as it can reflect patients’ thoughts or experiences more directly than quantitative research. Previous qualitative studies of MHD patients have focused mainly on symptoms, QOL perceptions of psychological problems, and related factors affecting hemodialysis specifically [31–34], but there are few qualitative studies on the mental health of MHD patients, making it difficult to identify its influencing factors and clarify the complex relationships between the different influencing factors. More qualitative studies are therefore required in order to better understand the mental health of MHD patients, as well as to be able to combine these findings with those of quantitative studies to offer more effective interventions to improve MHD patients’ mental health.

This qualitative study aimed to address these gaps in the current literature using semi-structured interviews to explore the mental health and its influencing factors of MHD patients. The explored factors could be used later as reference points when proposing clinical interventions to improve patients’ mental health. Emotional state and well-being were used as indicators to measure patients’ mental health.

## Methods

### Sampling

Participants were recruited through convenience sampling from the first author’s hospital between April and June 2022. The inclusion criteria were (1) patient was diagnosed with ESRD and had been on maintenance hemodialysis for more than three months; (2) patient age ≥ 18 years; (3) patient was receiving hemodialysis three times a week, for four hours each time. The exclusion criteria were (1) patient had severe complications or experienced major traumatic events (such as bereavement, divorce, unemployment) occurring within the previous three months; (2) patient was unconscious, was uncapable of normal language expression, and was unable to agree to sign the informed consent form. This study was conducted in accordance with the Helsinki Declaration and was approved by Zhanlanlu Hospital, Beijing, China.

## Study design and data collection

Each participant was invited to participate in a face-to-face semi-structured interview at the hemodialysis center on a routine hemodialysis day. Each participant signed an informed consent form and had completed a demographic survey before the interview began. They were each informed about the purpose of the study and the requirement that the interview be recorded.

The interview questions were based on a summary of previous literature [35–38]. Previous studies have identified several factors affecting different aspects of mental health, including complications affecting patients’ QOL [35], multiple stresses causing physical and mental burdens [36], perceived social support which had a significant impact on patients’ mental health [37], and acceptance of disease which played a particularly important role in the QOL and mental health of MHD patients [38]. Combined with the researchers’ experience with MHD patients and the review of previous studies, the interview questions focused on: (1) the MHD patient’s acceptance of their disease and hemodialysis treatment; (2) the physical changes they had experienced and the impact of these on the patient’s QOL; (3) their stress levels and emotional state, as well as their methods of stress release; (4) the patient’s feeling of well-being and what increased their level of happiness; (5) the social support the MHD patient experienced, particularly from their primary caregiver(s); and (6) their amount of help they expected to receive from medical staff. In this study, we used patients’ emotional state and well-being to assess their mental health.

Before beginning the formal interview part of the study, one trial interview was conducted to ensure the suitability of the questions and the length of time the interview would take. After this, the formal process began and interviews were scheduled. The formal interview lasted approximately 20 min and was recorded and transcribed verbatim using iFLYTEK Hearing software. After the interview had been processed, each transcription was proofread by two researchers independently.

## Data analyses

Following the principles of Grounded Theory [39], the sorted transcription documents were input into QSR International NVivo12 software for data analyses. First, nodes were created in the software to store ideas and excerpted content from the transcripts, with each node matching a memo. Second, initial coding took place after comparing the data. To improve the analytical reliability of the coding, another physician was invited to conduct independent coding of a sample of the transcripts (10% of the total transcription files). Third, data collection and analysis were conducted. Finally, constant comparative analysis and category identification of the interview content was completed. The guidelines for consolidated criteria for reporting qualitative research (COREQ) were followed during the whole study procedure [40].

## Results

### Sample description

A total of 35 MHD patients who met the participant inclusion criteria took part in the study, comprising 10 women (28.6%) and 25 men (71.4%). The mean age was 63.6 years.

Table 1 lists the demographic and clinical characteristics of the 35 participants.

**Table 1:** Demographic and clinical characteristics of the participants (N = 35)

Characteristics	n (%)
Gender	
Male	25 (71.4)
Female	10 (28.6)
Age (years)	
30–39	2 (5.7)
40–49	1 (2.9)
50–59	7 (20.0)
60–69	15 (42.9)
70–79	7 (20.0)
80–89	3 (8.5)
Primary disease	
Diabetic nephropathy	23 (65.7)
Chronic glomerulonephritis	6 (17.1)
Hyperuricemic nephropathy	3 (8.5)
ANCA-associated vasculitis	1 (2.9)
Polycystic kidney disease	1 (2.9)
Renal tumor	1 (2.9)
Months since receiving hemodialysis	
3–12 months	5 (14.3)
13–36 months	5 (14.3)
37–60 months	9 (25.7)
61–120 months	14 (40.0)
> 121 months	2 (5.7)

### MHD patients’ mental health and its influencing factors

Influencing factors on MHD patients’ mental health were recorded by examining the mental health history of each participant, starting from when they began to receive hemodialysis treatment up until the moment of the interview. Four themes had been identified to assess the diverse states of mental health: (1) acceptance of disease, (2) complications, (3) stress and coping styles, and (4) social support.

Examples of representative quotes are provided in Table 2

**Table 2:** Illustrative quotations for the four themes assessing mental health

Themes	Minor themes	Minimal themes	Quotes
Acceptance of disease	High acceptance group	Acceptance at the early stage of hemodialysis	“[…] My father had polycystic kidney disease, and he died of this disease. This is a genetic disease that I had long known I was at risk for. I was ready for it when I eventually learned that I also had this disease. The kidney transplant was not an option, so I knew that hemodialysis was the only way to stay alive […]” (MHD18, male, 67 years old, polycystic kidney disease)
			“[…] I had already died once, so I could accept anything. I underwent four surgeries under general anesthesia, included an amputation. Once, I was in a coma in the ICU for several days, and others thought I would not wake up, but I finally made it and was transferred to the normal ward. It took me a long time to recover and get out of the hospital, but I knew since then that I could accept anything. If you want to live, you have to do hemodialysis; if you don’t want to live, you don’t need to do hemodialysis. Why not? Hemodialysis is meant to keep you alive[…]” (MHD22, male, 54 years old, diabetic nephropathy)
			“[…] People should learn to be grateful. Although I’m sick and have suffered some pain, it is already a very good thing that I can have regular hemodialysis. I’m not afraid of death. All of this is arranged by God. God will always bless us! […]” (MHD20, female, 59 years old, kidney tumor)
		Gradually accepted disease and hemodialysis	“[…] When I first learned that hemodialysis was needed, I thought it was a big treatment and it caused me a certain degree of psychological stress. However, after routine hemodialysis, my physical condition has gradually normalized, which has greatly reduced my psychological stress. Now I can gradually accept my condition […]” (MHD2, male, 54 years old, diabetic nephropathy)
			“[…] In the past, my eyes could not see, my body was uncomfortable, and I could not take care of myself. When I first began hemodialysis, I felt depressed. Everyone advised me that I would be fine after hemodialysis. And, my body did become much better after hemodialysis, and I was able to see after the eye surgery. Now I can see things clearly and take care of myself in life. I can gradually accept the hemodialysis […]” (MHD9, male, 59 years old, diabetic nephropathy)
	Low acceptance group	Cannot accept disease and hemodialysis	“[…] Why me? I haven’t done anything wrong, and now I’m on hemodialysis. I can’t accept it, but there’s no other choice to stay alive […]” (MHD11, female, 74 years old, diabetic nephropathy)
			“[…] I used to be a math teacher. After the students had graduated, I found out that I was sick through a physical examination. At the time, I couldn’t accept it, that I would die soon. When I first started hemodialysis, I was very scared. Although I’m no longer afraid, I still can’t accept this reality. I’m very depressed since I can’t do anything now […]” (MHD32, male, 64 years old, diabetic nephropathy)
		Forced to accept disease and hemodialysis	“[…] I had been ill for many years. At first, I took medicine regularly, but gradually my kidney stopped working. I had to start hemodialysis. I did not know what hemodialysis was. I didn’t feel too depressed, just lived day by day, yielded to my fate […]” (MHD12, male, 87 years old, chronic glomerulonephritis)
			“[…] The disease cannot be cured, I can only maintain my life. I can’t understand why this is, but there is no other way, you and the disease have to coexist […]” (MHD16, male, 81 years old, hypertensive nephropathy)
Complications	Acute complications		“[…] Before the hemodialysis, I sometimes felt nauseated, nauseous, and itchy. Now, after a few months of regular hemodialysis, I feel less nauseous and the itchiness has stopped. In the middle and later stages of hemodialysis, though, I usually have a headache. And I’m very tired after the hemodialysis […]” (MHD32, male, 64 years old, diabetic nephropathy)
			“[…] After the hemodialysis, I felt very tired and very weak. When I got home, I would hurry to bed after eating. I wouldn’t recover until the next day, but I only ever took one day off. On the third day, I would have hemodialysis again. I was really irritable. Three times a week for hemodialysis is really a waste of time, which also seriously affects my quality of life […]” (MHD19, female, 65 years old, ANCA-associated vasculitis)
	Chronic complications		“[…] I didn’t feel anything else, but I was itchy, so itchy. I scratched all over my body. The itching seriously affected my quality of life. Last winter, I had pneumonia, and since then my legs have felt weak and I’ve found it very hard to walk […]” (MHD29, male, 79 years old, diabetic nephropathy)
			“[…] My body is definitely becoming weaker and weaker. It’s not like it used to be. I used to have a lot of ideas of things to do, but now I don’t have any ideas because of my body. My legs have become more and more weak, and my eyes cannot see anything, I dare not go out, in case I fall down accidentally […]” (MHD23, female, 54 years old, diabetic nephropathy)
			“[…] Sometimes my body is very uncomfortable, and all kinds of small problems appear. Sometimes I feel that I take too much medicine, and I don’t want to take it anymore. Vascular access complications severely affect my life […]” (MHD12, male, 87 years old, chronic glomerulonephritis)
Stress and coping styles	Self-perceived stress group		“[…] When I’m stressed and unhappy, I can go a day without talking or watching TV at home. I’m not interested in doing anything, including playing with my phone. In fact, as long as I don’t think about anything, it relieves the stress. If I don’t pay any attention to it, these emotions will pass after a while[…]” (MHD23, female, 52 years old, diabetic nephropathy).
			“[…] I have to undergo hemodialysis three times a week, and this is stressful and it makes me really irritable. My husband usually tells me, ‘You should accept your fate and leave the rest of your life to me. I will take good care of you, let’s eat whatever you want.’ It helps me let go of a lot of the stress. He sometimes takes me out for a drive to relax, I’m very happy for this […]” (MHD19, female, 65 years old, ANCA-associated vasculitis)
	Self-perceived stress-free group		“[…] I don’t experience any stress, and don’t need to worry about anything, Because I already understand my disease in its entirety. I feel good. The only thing I need to do is to take care of myself […]” (MHD18, male, 67 years old, polycystic kidney disease)
			“[…] I don’t feel any stress now, and I feel quite confident. I’m just going to live like this. I will do whatever the doctor says. I plan to live for another 20 years, and by then I will be in my 80s […]” (MHD25, male, 62 years old, chronic glomerulonephritis)
Social support	Family	Spouse	“[…] The person who takes care of me is my lover. She is very hardworking and helps me a lot. She takes care not only of me, but also my parents and my nephew. I am really grateful for her […]” (MHD2, male, 54 years old, diabetic nephropathy)
			“[…] Usually my husband does the housework. He usually asks me what I want to eat. If it isn’t easy for him to cook, he will go out and buy it. He usually buys my favorite food for me, which makes me very cared for. Sometimes my arms or legs hurt, and he will immediately give me massages. My daughter can’t take care of me because she has to work and she also has children to raise. She calls and says hello to me most of the time, but in my daily life, I depend primarily on my husband […]” (MHD19, female, 65 years old, ANCA-associated vasculitis)
		Children	“[…] My daughter-in-law takes charge of cooking for me, and my son usually drives me to the hospital. They all care about me. Every time I go to see a doctor, my son drives and his wife helps me by pushing the wheelchair and contacting the hospital […]” (MHD12, male, 87 years old, chronic glomerulonephritis)
		Parents	“[…] Usually, my parents take care of me, and urge me to take my medicine. I can drive to the hemodialysis appointments by myself […]” (MHD17, male, 33 years old, diabetic nephropathy)
		Relatives	“[…] My sister usually calls and chats with me, updates me, and gives me emotional support. My cousin drives me to the hospital every time […]” (MHD13, male, 56 years old, diabetic nephropathy)
	Friends		“[…} My family members are in other cities, and I am the only one working in Beijing. Right now I am in a good condition and can take care of myself. When I feel lonely, though, I usually invite several friends over to have dinner and chat. They make me happy […]” (MHD21, male, 33 years old, chronic glomerulonephritis)
	Medical staff		“[…] Now, the closest people to me are the doctors and nurses who arrange the hemodialysis for me, explain the tests to me, adjust my medicine, and usually chat with me. It’s all good now […]” (MHD16, male, 81 years old, hypertensive nephropathy)

### Theme 1: acceptance of disease

Acceptance of disease refers to a patient’s acceptance of their disease and hemodialysis treatment, which includes their capability of gradually adapting to living with the disease, receiving routine treatment for the disease, grasping the development process of the disease, and accepting the uncontrollable consequences of the disease. Patients were divided into two groups according to the degree of their current acceptance of disease during the interview: a high acceptance group and a low acceptance group. The high acceptance group included both patients who actively treated their disease at the beginning of hemodialysis, as well as those who had experienced a low degree of acceptance at the beginning of hemodialysis, but whose acceptance increased as their physical condition improved through medical treatments, thus, they experienced more positive emotions. Patients in the low acceptance group were characterized by living with negative emotions and who felt they were being forced to accept their disease and the necessary treatments against their desires.

Some of the MHD patients with a high acceptance of disease had a thorough understanding of their disease from an early stage of hemodialysis. These individuals showed a high degree of acceptance of disease and hemodialysis treatment, and actively cooperated with medical staff, following the medical instructions during hemodialysis treatment and drug adjustment, which meant that their physical conditions were more quickly stabilized. For the other patients in the high acceptance of disease group, although they initially had demonstrated a low degree of acceptance of the disease, after their physical conditions gradually improved through the hemodialysis and drug treatments, they began to accept their disease and the related treatments, including the hemodialysis. This transition improved their acceptance of the disease.

The MHD patients who accepted their disease at the early stage of hemodialysis could be divided into three categories. The first category was those living with familial diseases, who already had a relatively in-depth understanding of their diseases before receiving hemodialysis. It was thus easier for them to cooperate with medical staff when receiving medical treatments. The second category had an accepting attitude toward everything after having experienced general ups and downs in life, thus they also positively accepted the medical treatments. The third category demonstrated strong religious beliefs, believing that everything was arranged by God, therefore, they could accept their disease and follow medical instructions for treatment.

Patients who accepted their disease gradually throughout the process of hemodialysis were unable to have a high degree of acceptance of disease at the start of hemodialysis, and thus suffered a heavy psychological burden. Through explanation of and analysis of hemodialysis by medical staff and with the support of their families and friends, these patients were reluctantly able to accept a period of regular hemodialysis treatment. Once accompanied by their significantly improved physical conditions, these participants were able to truly accept their disease and began to accept the hemodialysis treatment and regular medication.

For the patients with a low acceptance of disease, the first category included those who understood they required hemodialysis treatment to stay alive, yet were still not able to accept this fact. They thought that fate was unfair and kept asking why they contracted this disease and needed hemodialysis treatment, while others did not. With this idea of the unfairness of it all forefront in their minds, despite requiring hemodialysis to survive, these patients demonstrated a low level of adherence to the treatment, and did not actively cooperate with medical staff. These patients were unable to psychologically take initiative to improve their acceptance of the disease.

The second category of patients with a low acceptance of disease said that they would yield to fate; they felt forced to accept their disease and the hemodialysis treatments to survive, but their attitude one of constant passivity and negativity. This hopeless attitude caused their emotional state to be terrible, and they would give up easily in the face of difficulty, and demonstrated a low degree of adherence to treatments. These patients often did not follow the advice received from medical staff, which led to a minimal or lack of improvement in their physical situation.

### Theme 2: complications

When assessing complications experienced by MHD patients, the probability of their occurrence depended on their age, primary disease, and years on hemodialysis. Elderly patients and patients who had been on hemodialysis for a longer period had a higher probability of contracting more acute or chronic complications. Furthermore, different primary diseases could lead to varying complications.

In the early stage of hemodialysis, MHD patients would often experience obvious improvements in their physical symptoms due to the rapid removal of excess toxins and volume load in the body. However, some patients might have also experienced acute complications during the hemodialysis process, thought these generally had a short duration and soon relieved. Acute complications can occur in the middle and late stages of the hemodialysis session, or even a few hours after the session has ended. During the interview, the acute complications mentioned most often by MHD patients included hypotension, muscle spasms, nausea, vomiting, headaches, and fatigue. Some of the long-term hemodialysis patients also experienced chronic complications, such as pruritus, cardiovascular complications, protein malnutrition, muscle weakness, or vascular access complications.

These acute or chronic complications were all associated with a low QOL. Moreover, patients who experienced more complications or more severe health conditions also experienced more frequent negative emotions as well as symptoms of anxiety and depression. Some of them suffered substantial pain because of these complications, causing them to lose any sense of happiness in anything.

### Theme 3: stress and coping styles

Most of the MHD patients undergoing routine hemodialysis experienced multiple forms of stress composed of varying degrees of physical, psychological, and economic pressures. This stress was closely related to their emotional state, in that as their stress level increased, their negative emotions would also increase, while their positive emotions would decrease. Some of the patients in the low acceptance of disease group tended towards regularly presenting obvious negative emotions, while patients with a higher degree of acceptance of disease and a more stable health condition tended to experience a lower level of stress and less emotional fluctuation, presenting higher appreciation for their current living conditions, family support, and medical conditions.

Different patients had quite different coping styles when it came to relieving stress. Patients with an optimistic attitude tended to adopt healthier coping methods, such as chatting with family members and friends, exercising, going for walks, gardening, singing, watching TV, reading, listening to music, or resting. In contrast, patients with a more pessimistic attitude tended to adopt more unhealthy coping methods such as smoking, drinking, or staying up late to play mahjong. Healthier coping styles tended to afford MHD patients more benefit in improving their physical condition, while unhealthy coping styles tended to increase the burden on patients’ physical fitness levels.

### Theme 4: social support

Social support was shown to play an important role in regulating the mental health of the MHD patients. During the interviews, patients reported that social support came primarily from family members, friends, and medical staff. Among these, family members were the ones most frequently involved in the MHD patients’ treatment and care – usually spouses, then followed by children. A few younger patients reported that their parents were their caregivers, and very few patients reported that they were taken care of by other relatives. Higher level of support from family members was related to both higher QOL and a better sense of well-being than that with lower level. Patients who lived alone were able to take care of themselves when they were in good physical condition, Though when they were in poor health they would hire a nurse or part-time worker for daily care. Patients’ friends mainly provided them with spiritual support, which included encouragement, sharing happiness, and visiting them to chat and engage with them. The MHD patients reported finding happiness through these interactions with their friends. Medical staff mainly provided patients with medical support, such as working with them to formulate treatment plans, diet and drug adjustments, exercise interventions, psychological counseling, offering them or educating them about new technologies and drugs, providing them with hemodialysis-related information, and so forth. Support from medical staff was also associated with patients’ good physical condition and quicker adaptation to accepting the disease and treatment.

## Discussion

This qualitative study, using a semi-structured interview format, aimed to explore the mental health and its influencing factors of MHD patients not currently undertaking interventional treatment in the hope of finding ways to improve their mental health. Based on Grounded Theory, semi-structured face-to-face interviews were conducted with 35 MHD patients, following the COREQ guidelines. Two indicators were used to assess MHD patients’ mental health, emotional state and well-being. All interviews were recorded and then analyzed using NVivo by two researchers, independently. Interview questions regarding the MHD patients’ state of mental health covered four themes: (1) acceptance of disease, (2) complications, (3) stress and coping styles, and (4) social support. Our findings suggest that all of these themes are significant influencing factors of MHD patients’ mental health. High degrees of acceptance of disease, healthy coping styles, and high levels of social support were positively correlated with MHD patients’ mental health. In contrast, low degrees of acceptance of disease, multiple complications, increased stress levels, and unhealthy coping styles were negatively correlated with MHD patients’ mental health.

For MHD patients, acceptance of disease was shown to play an important role in affecting their psychological state. The degree of one’s acceptance of disease could be changed, but the direction in which this attitude can change is not fixed. It is unclear when or whether MHD patients in the low acceptance group might shift into the high acceptance group, as there can be many influencing factors at play. Patients with a high degree of acceptance of disease often show more positive emotions. When acute or chronic complications occur, these patients tend to respond proactively, seeking solutions, asking for help from others, and cooperating with medical staff to address the problems. They also demonstrate a more optimistic attitude towards daily life. Despite being sick, they nonetheless still feel that their lives are no worse than those of others. Some patients are able to reduce their stress level and increase their positive emotions through healthy coping styles, such as moderate physical exercise. While undergoing treatment, they take medical staff seriously, and follow their advice while communicating well with their family members and friends. These positive activities bring these MHD patients a higher sense of well-being.

The findings of the current study are consistent with those of previous studies, in that the higher a patient’s acceptance of disease, the better their adherence to treatment and medication. The higher their degree of cooperation, the more stable their physical condition, and patients with a more stable condition will experience reduced self-perceived stress, improved QOL, and increased positive emotions [38, 41, 42] and well-being [43].

While undergoing treatment, MHD patients might experience both acute and chronic complications, which can cause intermittent or persistent physical discomfort and pain, which can lead to increased negative emotions [44]. The severity of these complications will affect the patient’s mental health both directly and indirectly [45]. Furthermore, studies have shown that an increased prevalence of complications is associated with a lower sense of well-being [45, 46]. For example, patients with systemic itching tend to demonstrate more obvious characteristics of a state of negative emotion than those who do not experience systemic itching [47].

Previous studies have also shown that the stress of MHD patients comes from numerous aspects, and is negatively correlated with their emotions, that is, the greater their stress, the more negative emotions they experience [48]. Other studies have shown a negative correlation between stress and well-being, with higher levels of stress resulting in lower levels of well-being [49]. In other words, reducing patients’ stress helps increase their positive emotions and sense of well-being.

Finally, previous research has found that patients with family members as part of their social support experience a high sense of well-being [50, 51]. Furthermore, patients with higher marital satisfaction experience less stress and feel more emotionally supported by their spouses [37]. Medical care provided by medical staff is also an influential part of a patient’s social support, meaning that the doctor-patient relationship plays a very important role in a patient’s sense of well-being [52]. When patients have more harmonious relationship with their doctor, they engage in more effective communication and cultivate mutual trust, thereby increasing the patient’s satisfaction and adherence to treatment and medication, reducing their negative emotions [53, 54].

## Limitations

This study does have several limitations. First, participants in this study came from only one hospital in Beijing, China, making this a single-center qualitative study. Thus, future research should use random sampling in a multi-center study design. Second, the interviews in this study employed a cross-sectional design. Dynamic changes in MHD patients’ level of acceptance of disease could not be observed during the single-time point interview. A longitudinal interview design should be considered in future studies to observe shifts in the relationship between changes in patients’ disease acceptance and level of mental health to further clarify the dynamic correlation between them.

## Conclusion

This study explored the mental health and its influencing factors of MHD patients. We found that acceptance of disease played an important role in the state of mental health of MHD patients, while the effects of complications, stress level, and coping styles, as well as the impact of social support were also shown to be related to MHD patients’ mental health. High acceptance of disease, healthy coping styles, and high social support were positively correlated with MHD patients’ mental health. Low acceptance of disease, multiple complications, increased stress, and unhealthy coping styles were negatively correlated with MHD patients’ mental health. The degree of acceptance of disease may be changed by the other three influencing factors. In future, more attention should be given to increasing MHD patients’ acceptance of disease to improve their mental health during hemodialysis treatment.

## Data Availability

The datasets generated and analyzed during the current study are not publicly available due to confidentiality and privacy related issues but are available from the corresponding author on reasonable request.

## Abbreviations

CKD: Chronic kidney disease
RRT: Renal replacement therapy
ESRD: End stage renal disease
QOL: Quality of life
MHD: Maintenance hemodialysis
WHO: World Health Organization
COREQ: Consolidated criteria for reporting qualitative research

## References

[1] El Nahas AM, Bello AK (2005). Chronic kidney disease: the global challenge. Lancet.

[2] Liyanage T, Ninomiya T, Jha V, Neal B, Patrice HM, Okpechi I (2015). Worldwide access to treatment for end-stage kidney disease: a systematic review. Lancet.

[3] Abdu A, Lawrence AB, Shuaibu AT, Sani T (2019). Hemodialysis outcome at Rasheed Shekoni Hospital. Nigerian J Basic Clin Sci.

[4] Chen TH, Wen YH, Chen CF, Tan AC, Chen YT, Chen FY (2020). The advantages of peritoneal dialysis over hemodialysis during the COVID-19 pandemic. Semin Dial.

[5] Chen LC, Tu IT, Yu IC, Tung TH, Huang HP, Lin YC (2022). The explorations of the awareness, contemplation, self-efficacy, and readiness of advance care planning, and its predictors in taiwanese patients while receiving hemodialysis treatment. BMC Palliat Care.

[6] Welte AL, Harpel T, Schumacher J, Barnes JL (2019). Registered dietitian nutritionists and perceptions of liberalizing the hemodialysis diet. Nutr Res Pract.

[7] Zhianfar L, Nadrian H, Shaghaghi A (2020). Enhancement of adherence to therapeutic and lifestyle recommendations among hemodialysis patients: an umbrella review of interventional strategies. Ther Clin Risk Manag.

[8] Wilund KR, Viana JL, Perez LM (2020). A critical review of exercise training in hemodialysis patients: personalized activity prescriptions are needed. Exerc Sport Sci Rev.

[9] Mali N, Ge J, Su F, Li C, Fan W (2022). Review of risk factors of malnutrition in maintenance hemodialysis patients. Arch Nephrol Urol.

[10] Balconi M, Angioletti L, De Filippis D, Bossola M (2019). Association between fatigue, motivational measures (BIS/BAS) and semi-structured psychosocial interview in hemodialytic treatment. BMC Psychol.

[11] Altınok Ersoy N, Akyar İ (2019). Multidimensional pruritus assessment in hemodialysis patients. BMC Nephrol.

[12] Slee A, McKeaveney C, Adamson G, Davenport A, Farrington K, Fouque D (2020). Estimating the prevalence of muscle wasting, weakness, and sarcopenia in hemodialysis patients. J Ren Nutr.

[13] Lyu B, Banerjee T, Scialla JJ, Shafi T, Yevzlin AS, Powe NR (2018). Vascular calcification markers and hemodialysis vascular access complications. Am J Nephrol.

[14] Joshi U, Subedi R, Poudel P, Ghimire PR, Panta S, Sigdel MR (2017). Assessment of quality of life in patients undergoing hemodialysis using WHOQOL-BREF questionnaire: a multicenter study. Int J Nephrol Renovasc Dis.

[15] Olczyk P, Kusztal M, Gołębiowski T, Letachowicz K, Krajewska M (2022). Cognitive impairment in end stage renal disease patients undergoing hemodialysis: markers and risk factors. Int J Environ Res Public Health.

[16] Semaan V, Noureddine S, Farhood L (2018). Prevalence of depression and anxiety in end-stage renal disease: a survey of patients undergoing hemodialysis. Appl Nurs Res.

[17] van Zwieten A, Wong G, Ruospo M, Palmer SC, Barulli MR, Iurillo A (2018). Prevalence and patterns of cognitive impairment in adult hemodialysis patients: the COGNITIVE-HD study. Nephrol Dial Transpl.

[18] Pojatić Đ, Tolj I, Pezerović D, Degmečić D (2021). Systematic review of alexithymia in the population of hemodialysis patients. J Clin Med.

[19] World Health Organization. The World Health Report 2001: Mental health: new understanding, new hope. 2001. https://apps.who.int/iris/bitstream/handle/10665/42390/WHR_2001.pdf?sequence=1&isAllowed=y.

[20] Keles B, McCrae N, Grealish A (2019). A systematic review: the influence of social media on depression, anxiety and psychological distress in adolescents. Int J Adolescence Youth.

[21] Rossell SL, Neill E, Phillipou A, Tan EJ, Toh WL, Van Rheenen TE, et al. An overview of current mental health in the general population of Australia during the COVID-19 pandemic: results from the COLLATE project. Psychiatry Res. 2021;296:Article113660. 10.1016/j.psychres.2020.113660. 10.1016/j.psychres.2020.113660 PMC7836867 33373808

[22] Fusar-Poli P, Salazar de Pablo G, De Micheli A, Nieman DH, Correll CU, Kessing LV (2020). What is good mental health? A scoping review. Eur Neuropsychopharmacol.

[23] Kocjan GZ, Kavčič T, Avsec A. Resilience matters: explaining the association between personality and psychological functioning during the COVID-19 pandemic. Int J Clin Health Psychol. 2021;21(1):Article 100198. 10.1016/j.ijchp.2020.08.002. 10.1016/j.ijchp.2020.08.002 PMC7753029 33363581

[24] Li C, Jiang S, Li N, Zhang Q (2018). Influence of social participation on life satisfaction and depression among chinese elderly: social support as a mediator. J Community Psychol.

[25] Trompetter HR, de Kleine E, Bohlmeijer ET (2017). Why does positive mental health buffer against psychopathology? An exploratory study on self-compassion as a resilience mechanism and adaptive emotion regulation strategy. Cogn Therapy Res.

[26] Khan A, Khan AH, Adnan AS, Sulaiman SAS, Mushtaq S (2019). Prevalence and predictors of depression among hemodialysis patients: a prospective follow-up study. BMC Public Health.

[27] Nadort E, Rijkers N, Schouten RW, Hoogeveen EK, Bos WJW, Vleming LJ, et al. Depression, anxiety and quality of life of hemodialysis patients before and during the COVID-19 pandemic. J Psychosom Res. 2022;158:Article 110917. 10.1016/j.jpsychores.2022.110917. 10.1016/j.jpsychores.2022.110917 PMC9008976 35462121

[28] Kubanek A, Paul P, Przybylak M, Kanclerz K, Rojek JJ, Renke M (2021). Use of sertraline in hemodialysis patients. Medicina.

[29] Ng CZ, Tang SC, Chan M, Tran BX, Ho CS, Tam WW, et al. A systematic review and meta-analysis of randomized controlled trials of cognitive behavioral therapy for hemodialysis patients with depression. J Psychosom Res. 2019;126:Article109834. 10.1016/j.jpsychores.2019.109834. 10.1016/j.jpsychores.2019.109834 31525637

[30] Beizaee Y, Rejeh N, Heravi-Karimooi M, Tadrisi SD, Griffiths P, Vaismoradi M (2018). The effect of guided imagery on anxiety, depression and vital signs in patients on hemodialysis. Complement Ther Clin Pract.

[31] Jacobson J, Ju A, Baumgart A, Unruh M, O’Donoghue D, Obrador G (2019). Patient perspectives on the meaning and impact of fatigue in hemodialysis: a systematic review and thematic analysis of qualitative studies. Am J Kidney Dis.

[32] Avdal EU, Ayvaz İ, Uran BNÖ, Yildirim JG, Sofulu F, Pamuk G (2020). Opinions of hemodialysis and peritoneum patients regarding depression and psychological problems which they experience: a qualitative study. J Infect Public Health.

[33] Hejazi SS, Hosseini M, Ebadi A, Alavi Majd H (2021). Components of quality of life in hemodialysis patients from family caregivers’ perspective: a qualitative study. BMC Nephrol.

[34] Kuo PY, Saran R, Argentina M, Heung M, Bragg-Gresham J, Krein S (2020). Cramping, crashing, cannulating, and clotting: a qualitative study of patients’ definitions of a “bad run” on hemodialysis. BMC Nephrol.

[35] Song JH. Complications of hemodialysis. In: Kim YL, Kawanishi H, editors. The essentials of clinical dialysis. Singapore: Springer; 2018. p. 105–126. 10.1007/978-981-10-1100-9_9.

[36] Ghaffari M, Morowatisharifabad MA, Mehrabi Y, Zare S, Askari J, Alizadeh S (2019). What are the hemodialysis patients’ style in coping with stress? A directed content analysis. Int J Community Based Nurs Midwifery.

[37] Jiang H, Wang L, Zhang Q, Liu D, Ding J, Lei Z (2015). Family functioning, marital satisfaction and social support in hemodialysis patients and their spouses. Stress Health.

[38] Marthoenis M, Syukri M, Abdullah A, Tandi TMR, Putra N, Laura H (2021). Quality of life, depression, and anxiety of patients undergoing hemodialysis: significant role of acceptance of the illness. Int J Psychiatry Med.

[39] Birks M, Mills J (2015). Grounded theory: a practical guide.

[40] Tong A, Sainsbury P, Craig J (2007). Consolidated criteria for reporting qualitative research (COREQ): a 32-item checklist for interviews and focus groups. Int J Qual Health Care.

[41] Gunarathne TGNS, Tang LY, Lim SK, Nanayakkara N, Damayanthi HDWT, Abdullah KL (2022). Factors associated with symptom burden in adults with chronic kidney disease undergoing hemodialysis: a prospective study. Int J Environ Res Public Health.

[42] Sawma T, Sanjab Y (2022). The association between sense of coherence and quality of life: a cross-sectional study in a sample of patients on hemodialysis. BMC Psychol.

[43] Tulip C, Fisher Z, Bankhead H, Wilkie L, Pridmore J, Gracey F (2020). Building wellbeing in people with chronic conditions: a qualitative evaluation of an 8-week positive psychotherapy intervention for people living with an acquired brain injury. Front Psychol.

[44] Chapman CR, Gavrin J (1993). Suffering and its relationship to pain. J Palliat Care.

[45] Wu Y-H, Hsu Y-J, Tzeng W-C (2022). Correlation between physical activity and psychological distress in patients receiving hemodialysis with comorbidities: a cross-sectional study. Int J Environ Res Public Health.

[46] Senmar M, Razaghpoor A, Mousavi AS, Zarrinkolah F, Esmaeili F, Rafiei H (2020). Psychological symptoms in patients on dialysis and their relationship with spiritual well-being. Florence Nightingale Journal of Nursing.

[47] Lee J, Suh H, Jung H, Park M, Ahn J (2021). Association between chronic pruritus, depression, and insomnia: a cross-sectional study. JAAD Int.

[48] Du J, Huang J, An Y, Xu W (2018). The relationship between stress and negative emotion: the mediating role of rumination. Clin Res Trials.

[49] Musa AS, Pevalin DJ, Al Khalaileh MA (2018). Spiritual well-being, depression, and stress among hemodialysis patients in Jordan. J Holist Nurs.

[50] Theodoritsi A, Aravantinou M-E, Gravani V, Bourtsi E, Vasilopoulou C, Theofilou P (2016). Factors associated with the social support of hemodialysis patients. Iran J Public Health.

[51] Kim YJ, Choi HJ (2012). The influence of uncertainty and social support on general well-being among hemodialysis patients. Korean J Rehabil Nurs.

[52] Allen D, Wainwright M, Hutchinson T (2011). Non-compliance’ as illness management: hemodialysis patients’ descriptions of adversarial patient–clinician interactions. Soc Sci Med.

[53] Martin LR, Williams SL, Haskard KB, DiMatteo MR. The challenge of patient adherence. Therapeutics and clinical risk management. 2005;1(3):189–199. https://doi.org/www.ncbi.nlm.nih.gov/pmc/articles/PMC1661624/. PMC1661624 18360559

[54] Hansen MS, Tesfaye W, Sewlal B, Mehta B, Sud K, Kairaitis L (2022). Psychosocial factors affecting patients with end-stage kidney disease and the impact of the social worker. J Nephrol.

